# A machine learning model predicting candidates for surgical treatment modality in patients with distant metastatic esophageal adenocarcinoma: A propensity score-matched analysis

**DOI:** 10.3389/fonc.2022.862536

**Published:** 2022-07-22

**Authors:** Fang Liao, Shuangbin Yu, Ying Zhou, Benying Feng

**Affiliations:** ^1^ Sichuan Provincial Center for Mental Health, Sichuan Academy of Medical Sciences and Sichuan Provincial People’s Hospital, Chengdu, China; ^2^ Key Laboratory of Psychosomatic Medicine, Chinese Academy of Medical Sciences, Chengdu, China; ^3^ Department of Medical Administration, Sichuan Academy of Medical Sciences and Sichuan People’s Hospital, Chengdu, China

**Keywords:** esophageal adenocarcinoma, overall survival, propensity score matching, metastasis, surgical treatment modality, machine learning

## Abstract

**Objective:**

To explore the role of surgical treatment modality on prognosis of metastatic esophageal adenocarcinoma (mEAC), as well as to construct a machine learning model to predict suitable candidates.

**Method:**

All mEAC patients pathologically diagnosed between January 2010 and December 2018 were extracted from the Surveillance, Epidemiology, and End Results (SEER) database. A 1:4 propensity score-matched analysis and a multivariate Cox analysis were performed to verify the prognostic value of surgical treatment modality. To identify suitable candidates, a machine learning model, classification and regression tree (CART), was constructed, and its predictive performance was evaluated by the area under receiver operating characteristic curve (AUC).

**Results:**

Of 4520 mEAC patients, 2901 (64.2%) were aged over 60 years and 4012 (88.8%) were males. There were 411 (9.1%) patients receiving surgical treatment modality. In the propensity score-matched analysis, surgical treatment modality was significantly associated with a decreased risk of death (HR: 0.47, 95% CI: 0.40-0.55); surgical patients had almost twice as much median survival time (MST) as those without resection (MST with 95% CI: 23 [17-27] months *vs.* 11 [11-12] months, *P <*0.0001). The similar association was also observed in the multivariate Cox analysis (HR: 0.47, 95% CI: 0.41-0.53). Then, a CART was constructed to identify suitable candidates for surgical treatment modality, with a relatively good discrimination ability (AUC with 95% CI: 0.710 [0.648-0.771]).

**Conclusion:**

Surgical treatment modality may be a promising strategy to prolong survival of mEAC patients. The CART in our study could serve as a useful tool to predict suitable candidates for surgical treatment modality. Further creditable studies are warranted to confirm our findings.

## Introduction

Esophageal cancer (EC) is a highly lethal cancer with the sixth most common cause of cancer-related death (1–3). Survival by stage is distinctly different for squamous cell carcinoma and adenocarcinoma, the two distinct histologic subtypes of EC. According to the AJCC guidelines, each major cell type is given its own section, with esophageal adenocarcinoma (EAC) representing the most common pathologic subtype in most Western populations, including the United States (4, 5). It is estimated that EAC will continuously increase in the incidence up to 2030, which certainly imposes heavy health and economic burdens (6). Nearly 30%- 40% of EC patients present metastases to distant lymph nodes or organs at the time of initial diagnosis (7). And such patients usually result in untoward outcomes, with the 5-year survival rate of lower than 5% (8, 9).

Principal treatment management for patients with distant metastases was generally limited to chemotherapy, radiation, as well as best supportive care, mainly depending on individuals’ clinical situation (10, 11). However, these treatment applications in such patients are largely palliative, with the main focus and goals of improving quality of life and reducing cancer-related symptoms. For example, creditable evidence is lacking regarding durable survival benefit from systemic chemotherapy, with the estimated median survival of less than 1 year (4).

In some recent studies with small sample sizes, it was reported that surgical treatment modality could lead to improvements in survival rate for selected metastatic patients (12, 13). Given the dismal prognosis, in the context of no effective treatment to prolong survival, surgical treatment modality may as well be a promising approach. Nevertheless, the retrospective observational researches might result in flawed results owing to the substantial unbalanced heterogeneity in baseline characteristics among such patients (7, 10, 14). Moreover, surgical treatment modality is not a first-line treatment for current patients with distant metastases. Just passable survival benefit would put patients in a dilemma, whether it is worthy to prolong survival rather than sustain the quality of life. A judicious method to predict candidates for surgical treatment modality is advisable, to gain better understanding and compliance from patients when potentially improving the survival.

Therefore, we conducted a 1:4 propensity score-matched analysis using a population-based cancer database to determine the role of surgical treatment modality on prognosis of mEAC. Also, a machine learning model was constructed to identify suitable candidates for surgical treatment modality.

## Methods

### Data source and patient selection

**Figure 1:**
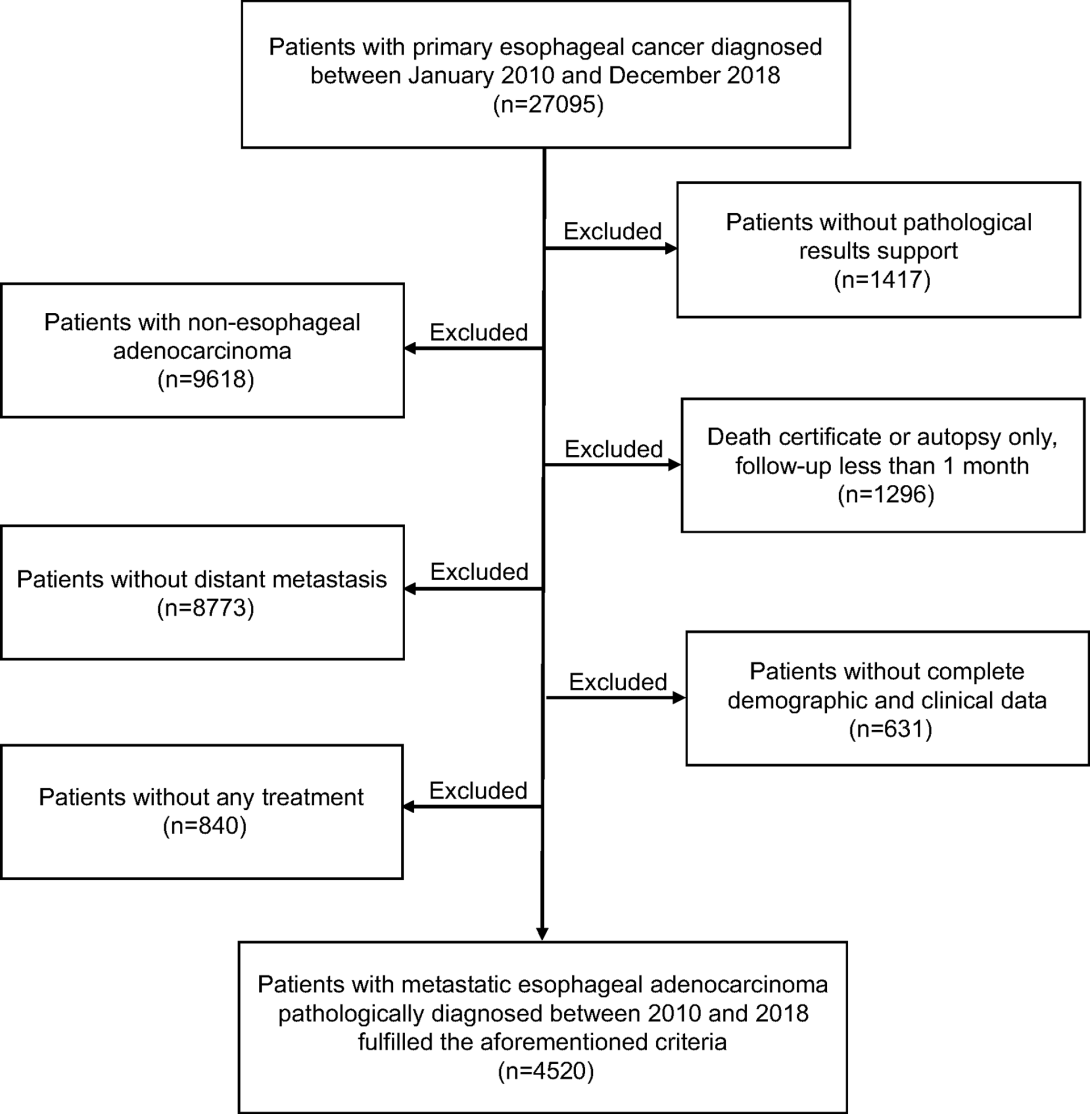
Flowchart showing detailed derivation of study population selection.

Data from the Surveillance, Epidemiology, and End Results (SEER) database, a nationwide cancer database covering nearly 30% of the US population, was used in the current study. All metastatic EAC patients pathologically identified as “one primary tumor only” during a period from January 2010 to December 2018 were considered eligible for our study. We selected these patients to generate a uniform dataset according to the eighth edition of American Joint Committee on Cancer (AJCC) staging system. Thus, for patients diagnosed before 2018, we manually translated 7th edition stages into their corresponding 8th edition stages. Cases identified by death certificate, autopsy only, or follow-up less than 1 month, lacked complete baseline information and those without receiving any treatment were excluded. The flowchart showing detailed derivation of study population selection is presented in **Figure 1**. This study was deemed exempt by the institutional review board (IRB), since all the extracted data were anonymous.

The demographic and clinicopathological characteristics of patients were extracted using the SEER*Stat software (version 8.3.9). In this study, marital status was reclassified as single (including a very low proportion of cases with unmarried or domestic partner), married, divorced/separated and widowed. All T stages were transformed into TX, T1, T2, T3 and T4, while all N stages were similarly redefined into NX, N0, N1, N2 and N3. Tumor grade was reclassified as well, moderate and poor (including a very low proportion of undifferentiated patients). Surgical treatment modality was categorized as any type of tumor-direct surgery (including local tumor excision, partial laryngectomy or total laryngectomy) with or without radiotherapy/chemoradiotherapy, while nonsurgical treatment modality was defined as radiotherapy, chemotherapy or chemoradiotherapy. Overall survival (OS) was employed as the primary end point, a period from the diagnosis to the death from any cause or last follow-up time.

### Statistical analysis

Baseline characteristics were presented as frequencies with percentages, and the distribution differences were compared by chi-square test. Propensity score matching (PSM) was adopted to reduce selection bias and imbalanced distributions of the confounding factors. Briefly, propensity score is the conditional probability of assignment to a particular treatment given a vector of observed covariates. In this study, to get a higher power of statistical test, surgical patients were matched 1:4 to those receiving non-surgical treatment modality in random order on the logit of the propensity score using the nearest-neighbor matching approach (maximum caliper distance, 0.03). The balance of covariates in both groups after PSM was assessed by calculating standardized mean differences (SMDs). A value of SMDs <10% was considered sufficiently balanced. Moreover, a multivariate Cox regression model was also constructed to assess the role of surgical treatment modality with adjustment for all the covariates included in the propensity analysis. Hazard ratios (HRs) was calculated with 95% confidence intervals (CIs). The OS of both groups was assessed by the Kaplan-Meier method and compared with the log-rank test.

We hypothesized that patients in the matched surgical cohort who survived longer than the lower limit value of the 95% CIs for median survival time (MST) would benefit from surgical treatment modality. Based on the above assumption, surgical patients were categorized as: suitable (benefit from surgery) *vs.* not-suitable (not benefit from surgery). Then, a machine learning model, classification and regression tree (CART), was constructed to identify suitable candidates for surgical treatment modality. In brief, CART could take all possible splits from all observed variables into consideration when split relative to an event, and select the variable that creates the most homogeneous clusters for the next split (15). In our study, the root node of CART contained all patients in the matched surgical cohort and was constantly separated into two subgroups utilizing the recursive iterative algorithm, until the survival rate of patients in the same subgroup was homogeneous. The prediction performance of the machine learning model was assessed by the area under the receiver operating characteristic (ROC) curve (AUC). All statistical analyses were performed with the software of R-4.0.2 (R Foundation, Vienna, Austria); a 2-sided *P*-value of < 0.05 was considered to be statistically significant.

## Results

### Patient demographics and characteristics

Totally, 4520 EAC patients with distant metastases were eligible for the study, of which 2901 (64.2%) were aged over 60 years and 4012 (88.8%) were males. There were 411 (9.1%) patients receiving surgical treatment modality. Of them, 38 cases underwent local tumor excision, 33 cases underwent partial esophagectomy and 340 cases underwent total esophagectomy. The median follow-up time was 8 months (interquartile range [IQR], 4 to 16 months). Patients’ demographic and clinicopathological characteristics are presented in **Table 1**. Except for sex, race and tumor site, the distributions of other characteristics (age, marriage, T stage, N stage, grade and metastatic sites) all showed significant differences between the surgical and non-surgical patients (all *P* < 0.05).

**Table 1:** Patients demographic characteristics and clinicopathological variables.

Characteristic	All patients(n=4520)	No. of patients (%)		*P* value
		Non-surgical (n=4109)	Surgical (n=411)	
Age, yr				<0.001
<60	1619 (35.8)	1431 (34.8)	188 (45.7)	
≥60	2901 (64.2)	2678 (65.2)	223 (54.3)	
Sex				0.239
Female	508 (11.2)	469 (11.4)	39 (9.5)	
Male	4012 (88.8)	3640 (88.6)	372 (90.5)	
Race				0.249
White	4239 (93.8)	3846 (93.6)	393 (95.6)	
Black	140 (3.1)	132 (3.2)	8 (1.9)	
Others	141 (3.1)	131 (3.2)	10 (2.4)	
Marital status				<0.001
Single	763 (16.9)	712 (17.3)	51 (12.4)	
Married	2880 (63.7)	2584 (62.9)	296(72.0)	
Divorced/separated	578 (12.8)	524 (12.8)	54 (13.1)	
Widowed	299 (6.6)	289 (7.0)	10 (2.4)	
T stage				<0.001
T1	751 (16.6)	714 (17.4)	37 (9.0)	
T2	257 (5.7)	215 (5.2)	42 (10.2)	
T3	1293 (28.6)	1046 (25.5)	247 (60.1)	
T4	682 (15.1)	629 (15.3)	53 (12.9)	
TX	1537 (34.0)	1505 (36.6)	32 (7.8)	
N stage				<0.001
N0	787 (17.4)	761 (18.5)	26 (6.3)	
N1	2377 (52.6)	2154 (52.4)	223 (54.3)	
N2	610 (13.5)	499 (12.1)	111 (27.0)	
N3	348 (7.7)	302 (7.3)	46 (11.2)	
NX	398 (8.8)	393 (9.6)	5 (1.2)	
Grade				0.001
Well	129 (2.9)	120 (2.9)	9 (2.2)	
Moderate	1365 (30.2)	1207 (29.4)	158 (38.4)	
Poor	2231 (49.4)	2042 (49.7)	189 (46.0)	
Unknown	795 (17.6)	740 (18.0)	55 (13.4)	
Site				0.293
Upper	38 (0.8)	36 (0.9)	2 (0.5)	
Middle	252 (5.6)	230 (5.6)	22 (5.4)	
Lower	3599 (79.6)	3257 (79.3)	342 (83.2)	
Overlap	230 (5.1)	211 (5.1)	19 (4.6)	
NOS	401 (8.9)	375 (9.1)	26 (6.3)	
Metastasis at bone				<0.001
No	3526 (78.0)	3129 (76.1)	397 (96.6)	
Yes	994 (22.0)	980 (23.9)	14 (3.4)	
Metastasis at brain				<0.001
No	4252 (94.1)	3846 (93.6)	406 (98.8)	
Yes	268 (5.9)	263 (6.4)	5 (1.2)	
Metastasis at liver				<0.001
No	2579 (57.1)	2204 (53.6)	375 (91.2)	
Yes	1941 (42.9)	1905 (46.4)	36 (8.8)	
Metastasis at lung				<0.001
No	3584 (79.3)	3191 (77.7)	393 (95.6)	
Yes	936 (20.7)	918 (22.3)	18 (4.4)	

### The prognostic role of surgical treatment modality *via* the PSM and multivariate Cox methods

**Figure 2:**
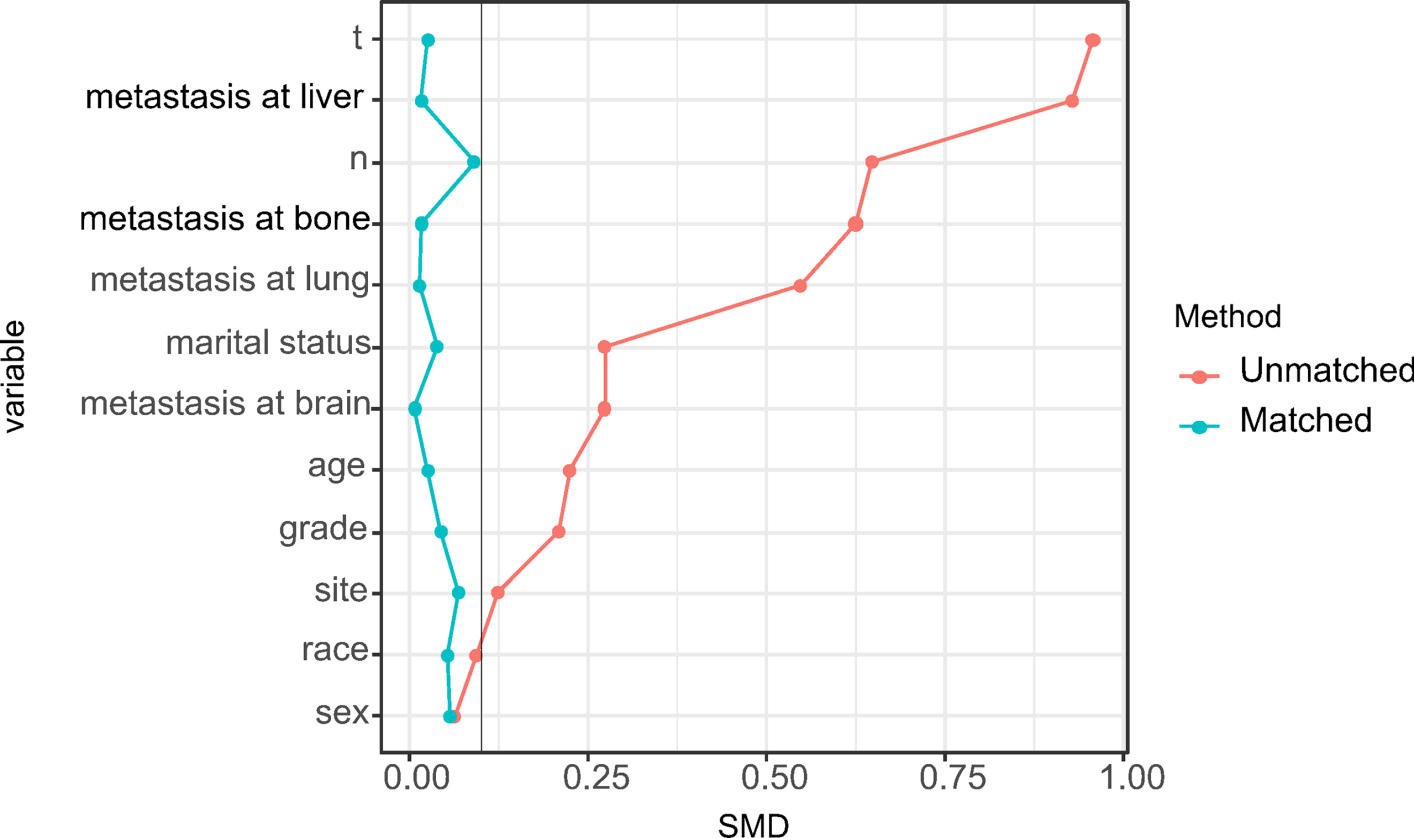
Distribution of standardized mean differences (SMDs) between the surgical and nonsurgical treatment modalities before and after propensity score matching.

**Figure 3:**
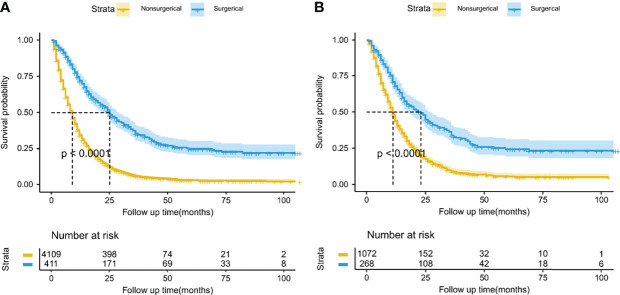
Kaplan-Meier overall survival curves between the surgical and nonsurgical treatment modalities before **(A)** and after **(B)** propensity score matching. The lightly colored width of the survival curve indicates the 95% confidence intervals of the Kaplan-Meier estimates.

Using PSM, there were 268 surgical patients matched to 1072 non-surgical patients with the ratio of 1:4; all the covariates in both groups were well balanced, with the highest SMD value of 9.1% for N stage (**Figure 2**). And we found surgical treatment modality was significantly associated with a decreased risk of death (HR: 0.47, 95% CI: 0.40-0.55; **Table 2**). In the multivariate Cox analysis, the risk of OS remained significantly less likely in surgical patients compared to non-surgical ones (HR: 0.47, 95% CI: 0.41-0.53). The Kaplan-Meier analysis also showed surgical patients had almost twice as much MST as those without resection (MST with 95% CI: 23 [17-27] months *vs.* 11 [11-12] months, *P <*0.0001, **Figure 3**).

**Table 2:** The role of different treatments on overall survival of patients with metastatic esophageal adenocarcinoma.

Variable	No. of Censored (%)	No. Of Death (%)	MST (95%CI)	*HR* (95%CI)
*Propensity Score-Matched Analysis*				
Treatment				
Non-surgical	195 (18.2)	877 (81.8)	11 (11-12)	1.00
Surgical	92 (34.3)	176 (65.7)	23 (17-27)	0.47 (0.40-0.55)
Multivariable Analysis^*^				
Treatment				
Non-surgical	620 (15.1)	3489 (84.9)	9 (8-9)	1.00
Surgical	148 (36.0)	263( 64.0)	25 (22-29)	0.47 (0.41-0.53)

^*^Adjustment for all the covariates included in the propensity analysis.

MST, median survival time; CI, confidence interval.

### CART to identify suitable candidates for surgical treatment modality

**Figure 4:**
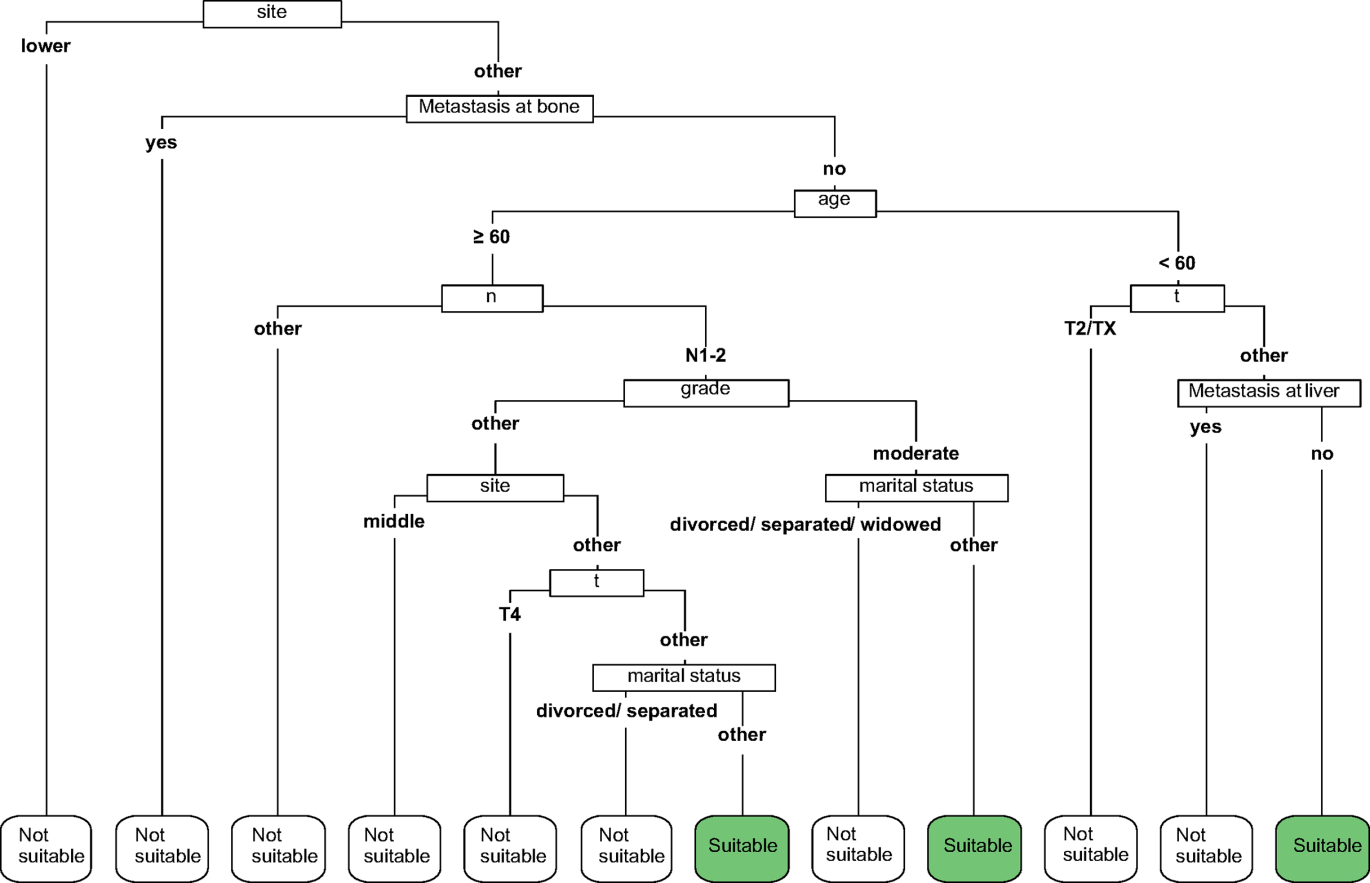
The classification and regression tree (CART) to select suitable candidates who would benefit from surgery.

**Figure 5:**
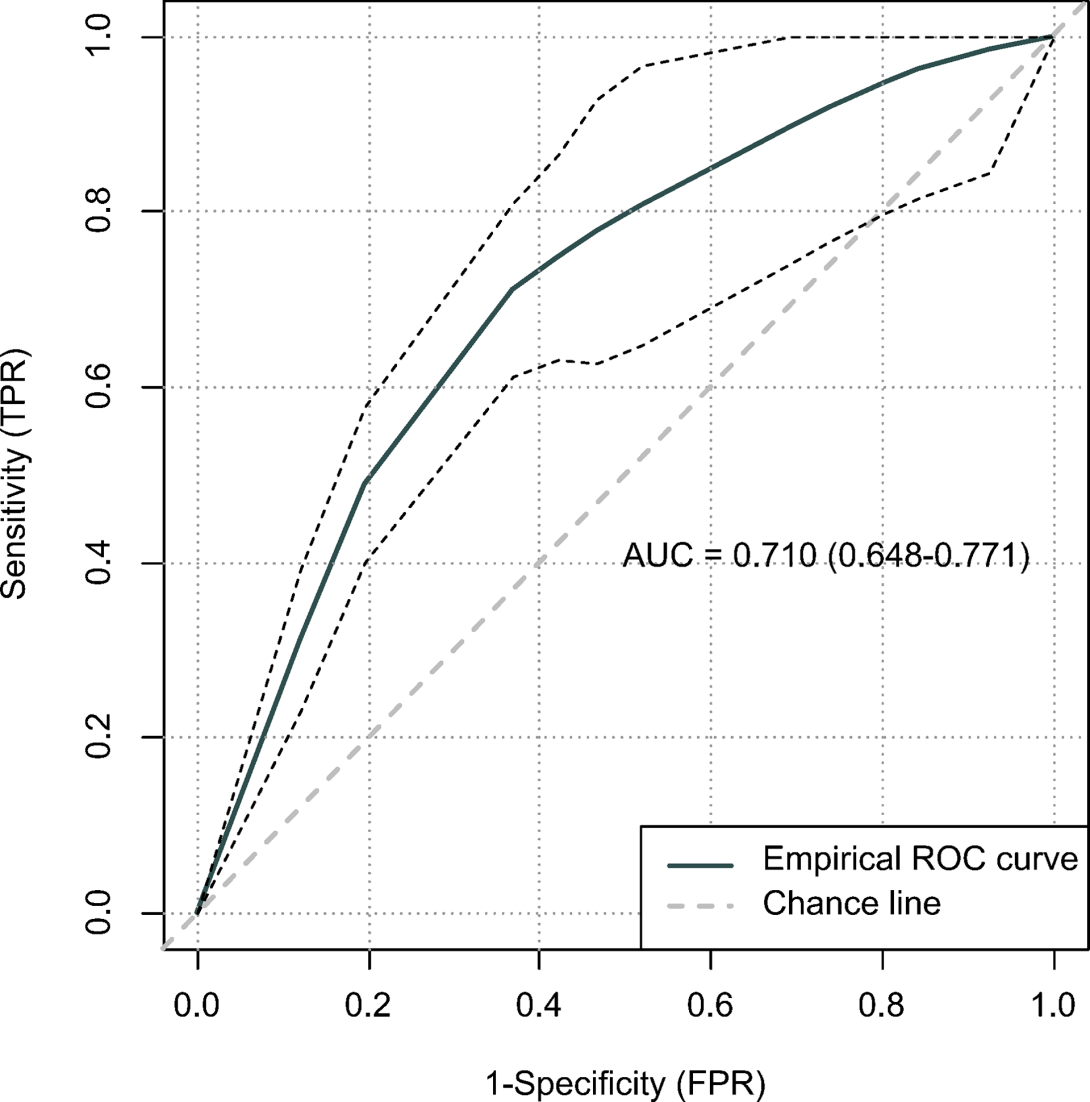
The predictive performance of the classification and regression tree (CART) using receiver operating characteristic (ROC) curve.

According to the lower limit value of the 95% CIs for MST, surgical patients in the matched surgical cohort were divided into 2 categories: 135 (50.4%) patients (survived ≥17 months) were classified as “suitable” while the remaining 133 (49.6%) cases (survived <17 months) were as “not-suitable”. Then, a machine learning model, CART, was constructed to identify suitable candidates for surgical treatment modality (**Figure 4**). The ROC curve showed CART possessed a relatively good discrimination ability to identify suitable candidates for surgical treatment modality (AUC with 95% CI: 0.710 [0.648-0.771]; **Figure 5**). In the Kaplan-Meier analysis, among all the 411 surgical patients, suitable candidates predicted by CART had a significantly better OS than not-suitable ones (*P <*0.0001; **Figure 1**).

## Discussion

Chemotherapy, radiation, as well as palliative supportive care seemed to be preferred for patients with mEAC by guidelines (16). The main cause lies in most mEAC patients are more willing to improve their quality of life and reduce cancer-related symptoms in the context that it is unclear whether surgical treatment modality would durably prolong survival. As such, in mEAC patients who receive surgical treatment modality, our understanding of disease related outcomes is still unclear, because of fairly limited cases.

Some previous researches revealed that no significant survival benefit was found from surgical treatment modality for metastatic EC patients (8, 17). Conversely, some researches indicated promising results on survival of metastatic EC patients after primary tumor resection (14, 18, 19). Other related studies also showed that multimodality therapy based on primary tumor surgery could prolong survival compared to single-modality treatment (20–22). These inconsistent observational results may be attributed to the relatively small sample sizes, or the substantial unbalanced heterogeneity in several critical baseline characteristics of metastatic EC patients, which need further verification by studies with large sample sizes using a more effective analytical method, such as propensity score-matched analysis (23). Moreover, according to the eighth edition of the AJCC staging system, classifications for esophageal squamous cell carcinoma and adenocarcinoma are no longer shared (24, 25). Therefore, it is more reasonable to discuss the effectiveness of surgical treatment modality separately according to different pathological types.

Based on clinical data from a large-scale nationwide cancer database, a 1:4 propensity score-matched analysis was utilized to explore the role of surgical treatment modality on prognosis of mEAC. In this study, all characteristics linked to patient survival, such as age, marital status, grade, T/N stage based on the 8th edition staging system, were replaced by a single composite propensity score to sufficiently balance the potential confounding. Notably, of mEAC patients receiving surgical treatment modality in our study, 340 (82.7%) cases underwent total esophagectomy. Our results found that surgical treatment modality in mEAC patients was significantly associated with an OS benefit. To give confidence to the robustness of our results, a multivariate Cox analysis in all mEAC patients which adjusted for all the covariates included in the propensity analysis was also conducted. The consistent results were still observed, indicating the evidence was creditable with respect to the role of surgical treatment modality on mEAC prognosis.

Since surgical treatment modality was not usually recommended for mEAC patients, they may hesitate whether it is worthy to receive primary tumor resection at the potential cost of bearing painful surgery-related side effects. In order to improve patients’ compliance, we hypothesized that a patient would benefit from surgical treatment modality if he/she survived longer than 17 months, a lower limit value of the 95% CIs for MST, which was almost twice as much as survival time of those without resection in the entire cohort. Then, a machine learning model was constructed to identify suitable candidates according to the cut-off value of 17 months. Given a relatively good discrimination ability was achieved (AUC= 0.710), our CART may serve as a useful tool to predict candidates who would benefit from surgical treatment modality.

There are also several limitations that need to be recognized in the study. First, it is regrettable that the SEER database could not provide information on overall comorbidity, performance status or detailed description on the surgical procedure, thus we failed to reduce this kind of unbalanced heterogeneity. Nevertheless, we excluded those patients without receiving any type of treatment, which may to some extent control the potential confounding. Second, more treatment details of mEAC patients were not available, it is unknown whether primary tumor surgery before/after the systemic treatment could enhance more survival benefit. Third, because of the limited number of surgical patients with mEAC, an internal validation was not performed for our CART, thus our model needs to be further confirmed in the future.

## Conclusion

The present study suggests that surgical treatment modality in mEAC patients has been positively associated with an OS benefit. Moreover, the CART may serve as a useful tool to predict suitable candidates who would benefit from surgical treatment modality. Further creditable studies are warranted to confirm our findings.

## Data availability statement

Publicly available datasets were analyzed in this study. This data can be found here: https://seer.cancer.gov/.

## Author contributions

FL had full access to all of the data in the study and takes responsibility for the integrity of the data and the accuracy of the data analysis. Concept and design: FL. Acquisition, analysis, or interpretation of data: FL, SY. Statistical analysis: FL, YZ. Drafting of the manuscript: FL, SY. Supervision: BFeng. All authors contributed to the article and approved the submitted version.

## Conflict of interest

The authors declare that the research was conducted in the absence of any commercial or financial relationships that could be construed as a potential conflict of interest.

## Publisher’s note

All claims expressed in this article are solely those of the authors and do not necessarily represent those of their affiliated organizations, or those of the publisher, the editors and the reviewers. Any product that may be evaluated in this article, or claim that may be made by its manufacturer, is not guaranteed or endorsed by the publisher.

## References

[1] Sung H Ferlay J Siegel RL Laversanne M Soerjomataram I Jemal A. Global cancer statistics 2020: GLOBOCAN estimates of incidence and mortality worldwide for 36 cancers in 185 countries. CA Cancer J Clin (2021) 71(3):209–49. doi: 10.3322/caac.21660 33538338

[2] Rustgi AK El-Serag HB. Esophageal carcinoma. N Engl J Med (2014) 371(26):2499–509. doi: 10.1056/NEJMra1314530 25539106

[3] Shao Y Geng Y Gu W Ning Z Huang J Pei H. Assessment of lymph node ratio to replace the pN categories system of classification of the TNM system in esophageal squamous cell carcinoma. J Thorac Oncol (2016) 11(10):1774–84. doi: 10.1016/j.jtho.2016.06.019 27393473

[4] van Rossum PSN Mohammad NH Vleggaar FP van Hillegersberg R. Treatment for unresectable or metastatic oesophageal cancer: Current evidence and trends. Nat Rev Gastroenterol Hepatol (2018) 15(4):235–49. doi: 10.1038/nrgastro.2017.162 29235549

[5] Shah MA Kennedy EB Catenacci DV Deighton DC Goodman KA Malhotra NK. Treatment of locally advanced esophageal carcinoma: ASCO guideline. J Clin Oncol (2020) 38(23):2677–94. doi: 10.1200/JCO.20.00866 32568633

[6] Zhang YH Guo LJ Yuan XL Hu B. Artificial intelligence-assisted esophageal cancer management: Now and future. World J Gastroenterol (2020) 26(35):5256–71. doi: 10.3748/wjg.v26.i35.5256 PMC7504247 32994686

[7] Xu J Lu D Zhang L Li J Sun G. Palliative resection or radiation of primary tumor prolonged survival for metastatic esophageal cancer. Cancer Med (2019) 8(17):7253–64. doi: 10.1002/cam4.2609 PMC6885868 31612596

[8] Tanaka T Fujita H Matono S Nagano T Nishimura K Murata K. Outcomes of multimodality therapy for stage IVB esophageal cancer with distant organ metastasis (M1-org). Dis Esophagus (2010) 23(8):646–51. doi: 10.1111/j.1442-2050.2010.01069.x 20545979

[9] Turgeman I Ben-Aharon I. Evolving treatment paradigms in esophageal cancer. Ann Transl Med (2021) 9(10):903. doi: 10.21037/atm.2020.03.110 34164537 PMC8184467

[10] Wu SG Xie WH Zhang ZQ Sun JY Li FY Lin HX. Surgery combined with radiotherapy improved survival in metastatic esophageal cancer in a surveillance epidemiology and end results population-based study. Sci Rep (2016) 6:28280. doi: 10.1038/srep28280 27323696 PMC4915008

[11] Fatehi Hassanabad A Chehade R Breadner D Raphael J. Esophageal carcinoma: Towards targeted therapies. Cell Oncol (Dordr) (2020) 43(2):195–209. doi: 10.1007/s13402-019-00488-2 31848929 PMC12990719

[12] Blank S Lordick F Dobritz M Grenacher L Burian M Langer R. A reliable risk score for stage IV esophagogastric cancer. Eur J Surg Oncol (2013) 39(8):823–30. doi: 10.1016/j.ejso.2013.01.005 23375470

[13] Yamashita K Katada N Moriya H Hosoda K Sakuramoto S Kikuchi S. Multimodality treatment and prognosis in esophageal squamous cell carcinoma requiring esophagectomy. Hepatogastroenterology (2014) 61(132):1042–8. 26158163

[14] Liu Z Zhang X Li B Jiang H Yang Y Hua R. A population-based predictive model predicting candidate for primary tumor surgery in patients with metastatic esophageal cancer. J Thorac Dis (2021) 13(2):870–82. doi: 10.21037/jtd-20-2347 PMC7947545 33717560

[15] Breiman L. Random forests. Mach Learning (2001) 45(1):5–32. doi: 10.1023/A:1010933404324

[16] Ku GY. Systemic therapy for esophageal cancer: Chemotherapy. Chin Clin Oncol (2017) 6(5):49. doi: 10.21037/cco.2017.07.06 29129089

[17] Saddoughi SA Reinersman JM Zhukov YO Taswell J Mara K Harmsen SW. Survival after surgical resection of stage IV esophageal cancer. Ann Thorac Surg (2017) 103(1):261–6. doi: 10.1016/j.athoracsur.2016.06.070 27623270

[18] Zhang R Zou J Li P Li Q Qiao Y Han J. Surgery to the primary tumor is associated with improved survival of patients with metastatic esophageal cancer: Propensity score-matched analyses of a large retrospective cohort. Dis Esophagus (2020). doi: 10.1093/dote/doz051 31175353

[19] Tokairin Y Kumagai Y Yamazaki S. [A case of postoperative liver metastasis of esophageal cancer remains in progression free after successfully resected]. Gan To Kagaku Ryoho (2009) 33(3):1–9. 20037456

[20] Wang J Suri JS Allen PK Liao Z Komaki R Ho L. Factors predictive of improved outcomes with multimodality local therapy after palliative chemotherapy for stage IV esophageal cancer. Am J Clin Oncol (2016) 39(3):228–35. doi: 10.1097/COC.0000000000000066 24710122

[21] Chao YK Wu YC Liu YH Tseng CK Chang HK Hsieh MJ. Distant nodal metastases from intrathoracic esophageal squamous cell carcinoma: Characteristics of long-term survivors after chemoradiotherapy. J Surg Oncol (2010) 102(2):158–62. doi: 10.1002/jso.21588 20648587

[22] Mudan SS Giakoustidis A Giakoustidis D Slevin M. Synchronous oesophagectomy and hepatic resection for metastatic oesophageal cancer: Report of a case. Hippokratia (2010) 14(4):291–3. PMC3031329 21311643

[23] Morgan CJ. Reducing bias using propensity score matching. J Nucl Cardiol (2018) 25(2):404–6. doi: 10.1007/s12350-017-1012-y 28776312

[24] Rice TW Patil DT Blackstone EH. 8th edition AJCC/UICC staging of cancers of the esophagus and esophagogastric junction: Application to clinical practice. Ann Cardiothorac Surg (2017) 6(2):119–30. doi: 10.21037/acs.2017.03.14 PMC5387145 28447000

[25] Raja S Ahmad U. Is newer actually better? where does the 8th edition outperform the 7th edition of the esophageal TNM staging system? Ann Surg Oncol (2021) 28(2):596–7. doi: 10.1245/s10434-020-09199-7 33090287

